# New ex vivo reporter assay system reveals that σ factors of an unculturable pathogen control gene regulation involved in the host switching between insects and plants

**DOI:** 10.1002/mbo3.93

**Published:** 2013-05-31

**Authors:** Yoshiko Ishii, Shigeyuki Kakizawa, Kenro Oshima

**Affiliations:** Department of Agricultural and Environmental Biology Graduate School of Agricultural and Life Sciences, The University of TokyoTokyo, Japan

**Keywords:** Host switching between plants and insects, new reporter assay system, sigma factors, transcriptional regulation, unculturable bacterial pathogen

## Abstract

Analysis of the environmental regulation of bacterial gene expression is important for understanding the nature, pathogenicity, and infection route of many pathogens. “*Candidatus* Phytoplasma asteris”, onion yellows strain M (OY-M), is a phytopathogenic bacterium that is able to adapt to quite different host environments, including plants and insects, with a relatively small ∼850 kb genome. The OY-M genome encodes two sigma (σ) factors, RpoD and FliA, that are homologous to *Escherichia coli* σ^70^ and σ^28^, respectively. Previous studies show that gene expression of OY-M dramatically changes upon the response to insect and plant hosts. However, very little is known about the relationship between the two σ factors and gene regulatory systems in OY-M, because phytoplasma cannot currently be cultured in vitro. Here, we developed an *E**scherichia* *c**oli*-based ex vivo reporter assay (EcERA) system to evaluate the transcriptional induction of phytoplasmal genes by the OY-M-derived σ factors. EcERA revealed that highly expressed genes in insect and plant hosts were regulated by RpoD and FliA, respectively. We also demonstrated that *rpoD* expression was significantly higher in insect than in plant hosts and *fliA* expression was similar between the hosts. These data indicate that phytoplasma-derived RpoD and FliA play key roles in the transcriptional switching mechanism during host switching between insects and plants. Our study will be invaluable to understand phytoplasmal transmission, virulence expression in plants, and the effect of infection on insect fitness. In addition, the novel EcERA system could be broadly applied to reveal transcriptional regulation mechanisms in other unculturable bacteria.

Phytoplasma, an unculturable plant pathogen, could infect plant and insect cells, and dramatically changes their genes upon the response to these hosts. By a new system developed in this study, interactions between phytoplasma promoters and sigma factors were analyzed, and overall gene expression regulation mechanism could be revealed. This model illustrates the RpoD and FliA regulatory network in phytoplasma cells during host switching.

## Introduction

Bacteria survive in diverse environments, such as the mammalian gut, seawater, and soil, by harboring mechanisms that sense changes in nutrient availability, osmolarity, temperature, and other external factors. This allows them to adapt to diverse environments by turning on and off specific sets of stress-response genes (Gottesman 1984). RNA polymerase (RNAP) plays a key role in regulating global gene expression pattern changes by rapidly modulating its promoter selectivity. Bacterial RNAP consists of five subunits, α_2_ββ’ω, that comprise the core enzyme (Browning and Busby 2004). Although the core RNAP alone can synthesize RNA, association with an accessory sigma (σ) subunit to form the RNAP holoenzyme is required to recognize the specific promoters (Browning and Busby 2004); this has been demonstrated by replacing the σ subunit on the core enzyme leading to changes in RNAP promoter selectivity (Browning and Busby 2004). In general, bacteria that can survive in varied environments contain many σ factors (Gruber and Gross 2003), which is probably due to the requirement of a large repertoire of regulatory mechanisms to adjust their metabolism to respond to varied environments (Gruber and Gross 2003). For example, *Mycoplasma genitalium*, an obligate cellular parasite, contains only one σ factor (Fraser et al. 1995), while *Escherichia coli*, a free-living organism, contains seven σ factors (Jishage et al. 1996).

Phytoplasmas are phytopathogenic bacteria that cause disease in many plants and crops, which dramatically decreases agricultural productivity (Weintraub and Beanland 2006). These bacteria are remarkable in their ability to adapt to drastically different hosts: plants and insects (Hogenhout et al. 2008). The bacteria inhabit phloem sieve elements in infected plants, and are transmitted by sap-sucking insect vectors that lead to disease dissemination (Lee and Davis 1992). Interestingly, phytoplasma infection induces vastly different effects in each host; although phytoplasma induces morphological abnormalities to plant host such as virescence, yellowing, phyllody, stunting, proliferation, and witches’ broom symptoms, they significantly increase the longevity and offspring number of insect hosts (Beanland et al. 2000; Hogenhout et al. 2008). Phytoplasmas are able to perform the complex events required for this host switching even with their small ∼850-kb genome. Four phytoplasma genomes have been completely sequenced (Oshima et al. 2004; Bai et al. 2006; Kube et al. 2008; Tran-Nguyen et al. 2008), and the number of genes involved in metabolism and other basic processes were found to be greatly reduced, which is similar to other bacteria having obligate associations with their hosts (Moran and Plague 2004).

We have previously demonstrated that “*Candidatus* Phytoplasma asteris,” onion yellows strain (OY-M) dramatically changes its own gene expression during the host switching between plant and insect hosts (Oshima et al. 2011). Analysis of these gene regulation mechanisms will be important to understand their host-adaptation mechanisms, infection strategies, and pathogenicity. Two σ factors, RpoD and FliA, were identified in all of the sequenced phytoplasma genomes (Oshima et al. 2004; Bai et al. 2006; Kube et al. 2008; Tran-Nguyen et al. 2008). RpoD has a high sequence similarity with the housekeeping σ^70^ factor and is encoded as a single copy gene in the phytoplasma genome. FliA is an alternative σ factor similar to σ^28^, and is encoded as multicopy genes within the gene clusters called potential mobile units (PMU) in the phytoplasma genome (Bai et al. 2006; Arashida et al. 2008). FliA-mediated transcription in most bacteria is associated with a stress response and/or with flagellar biosynthesis (Kazmierczak et al. 2005). Furthermore, PMU-encoded genes were believed to contribute to phytoplasma host adaptation (Toruño et al. 2010). Thus, these σ factors likely play key roles in the regulation of gene expression during host switching between insects and plants. However, this mechanism is not well understood because phytoplasmas currently cannot be cultured, which makes it difficult to analyze the σ factors of phytoplasma at molecular biological level.

In this study, we determined the intracellular *rpoD* and *fliA* mRNA levels by quantitative real-time reverse-transcription polymerase chain reaction assay (qRT-PCR) after OY-M infection between insect and plant hosts. We then developed a novel approach called the “*E**scherichia* *c**oli*-based ex vivo reporter assay” (EcERA) system to evaluate interaction between phytoplasmal promoters and σ factors. We demonstrate that RpoD and FliA regulate genes significantly expressed in insect and plant hosts, respectively. These findings help to clarify the phytoplasma transcriptional regulation during host switching. In addition, the new assay system established in this study could be applied to further understand the transcriptional regulation of other unculturable bacteria, such as important environmental or commensal bacteria.

## Experimental Procedures

### Phytoplasma lines and growth conditions

The “*Candidatus* Phytoplasma asteris” OY strain (OY) was isolated in Saga Prefecture, Japan (Shiomi et al. 1996). One derivative line (OY-M) was maintained in garland chrysanthemum (*Chrysanthemum coronarium*), using the leafhopper vector insect *Macrosteles striifrons* (Oshima et al. 2001). Plants infected with OY-M produce many lateral shoots, but exhibit only mild leaf yellowing and almost no stunting. OY-M-infected host plants exhibiting typical symptoms were maintained at 25°C in a greenhouse with a 16-h light/8-h dark photoperiod until they were used for analysis. OY-M-carrying leafhoppers that fed on OY-M-infected plants for 40 days were used. Healthy plants and non-OY-M-carrying leafhoppers were used as negative controls.

### RNA extraction

ISOGEN reagent (Nippon Gene, Tokyo, Japan) was used to isolate total RNAs from OY-M-infected insects (*M. striifrons*) and plants (*C. coronarium*) following the manufacturer's instructions. To eliminate DNA contamination, total RNAs were treated with DNase I (Takara, Shiga, Japan) following the manufacturer's instructions. RNA was quantified by UV spectrophotometry and analyzed using a 1% agarose gel to ensure RNA integrity before use.

### Relative gene expression quantification by qRT-PCR

#### Construction of cDNA standards

Total RNA from OY-M-infected insects was reverse-transcribed using the High-Capacity cDNA Reverse Transcription Kit (Applied Biosystems, Piscataway, NJ) according to the manufacturer's instructions. Serial 10-fold dilutions of the cDNA were prepared to compare relative expression levels of the OY-M-derived *rrnB, rpsJ, gyrB, PAM289, mdlB, hflB, himA,* and *dam* genes between insect and plant hosts.

#### Quantitative real-time RT-PCR

Total RNA samples from OY-M-infected insects and plants were reverse-transcribed using the High-Capacity cDNA Reverse Transcription Kit (Applied Biosystems) according to the manufacturer's instructions. For qRT-PCR, primer sets were designed from the OY-M genome (Acc. No. AP006628) using the Primer Express software (Applied Biosystems) (Table S2). qRT-PCR assays with experimental samples, calibration standards, or negative controls were performed using the Thermal Cycler Dice Real Time System (Takara) with *tufB* (translation elongation factor EF-Tu gene) as an internal standard. Briefly, the 20-μL reaction mixture consisted of 5 μL of diluted cDNA, 10 μL of SYBR Premix Ex Taq II (Takara), 1.2 μL each of the 5-μmol/L forward and reverse primers, and 2.6 μL of nuclease-free water. The cycling conditions consisted of a 10-min denaturation step at 95°C to activate the hot-start polymerase, followed by 50 cycles of 5-sec denaturation step at 95°C and 30-sec annealing and extension step at 60°C. Melting curve was obtained following a denaturation period of 15 sec at 95°C, starting at 65°C and ending at 95°C, to assess the specificity of qRT-PCR products. Using total RNAs from OY-M-infected insects and plants, each individual OY-M gene measurement was repeated at least three times. Relative expression levels in each sample were calculated with the Thermal Cycler Dice Real Time System Software (version 4.00) (Takara) based on the respective standard curves for each gene using a 10-fold dilution series. Results are expressed as the mean ± standard error of the mean (SE). Significant differences between the mean values were evaluated by Student's *t*-test with Statcel software (OMS Publishing, Saitama, Japan).

We previously performed several experiments to confirm tufB as internal control. Expression levels of several OY-M genes were compared between plants and insects with three housekeeping genes tufB, RpsP (ribosomal protein, small subunit P), and Ung (uracil-DNA glycosylase), as internal controls, and same results were obtained between controls (data not shown). While expression levels of these three genes were compared using one of three genes as a control, expression levels of all three genes were not different between plants and insects (data not shown). TufB gene was also used as a internal control in our previous paper (Oshima et al. 2011).

### Absolute quantification of gene expression by qRT-PCR

#### In vitro transcription

Fragments of *rpoA, rpoD, fliA,* and *tufB* were amplified using PCR primers (Table S1) and were cloned into pBlueScript II SK (+) vectors (Fermentas, Vilnius, Lithuania). The clones were used for in vitro transcription with the T7 RNAP (Ambion, Austin, TX) to generate in vitro transcribed RNA. For this purpose, the recombinant plasmids were first linearized by digestion with the *Sac*I enzyme (Takara, Shiga, Japan). In vitro transcription from linearized plasmid DNAs was then carried out using MEGAscript T7 Kit (Ambion), and the transcribed RNA was treated with DNase I (Takara) following the manufacturer's instructions. RNA was purified using the RNeasy kit (Qiagen, Hilden, Germany), and was eluted in nuclease-free water. RNA concentration was determined by UV spectrophotometry and analyzed using a native 1% agarose gel to ensure RNA integrity before use.

#### Construction of in vitro transcribed RNA standards

The concentration of in vitro transcribed *rpoA, rpoD, fliA,* and *tufB* RNAs was measured by UV spectrophotometry, and the absolute number of molecules was calculated as described by Fronhoffs et al. (2002). Briefly, serial 10-fold dilutions of each RNA were reverse-transcribed using the High-Capacity cDNA Reverse Transcription Kit (Applied Biosystems) according to the manufacturer's instructions, and the absolute quantities of *rpoD, fliA, rpoA*, and *tufB* in the OY-M-infected insects and plants were calculated.

#### Quantitative real-time RT-PCR

qRT-PCR assays were performed as previously described. Each individual OY-M gene measurement was repeated at least 8 times. Absolute number of OY-M gene molecules (*rpoA, rpoD, fliA,* and *tufB*) in total RNAs from OY-M-infected insects and plants were calculated with Thermal Cycler Dice Real Time System Software (version 4.00; Takara) based on the respective standard curves for each gene using a 10-fold dilution series. Results are expressed as the mean ± SE. Significant differences between the mean values were evaluated by Scheffe's test with Statcel software (OMS Publishing).

### Molecular cloning and plasmid construction

The plasmids used in this study are listed in Table 1. Oligonucleotide primers containing target sequences and cloning sites were synthesized by Operon Biotechnologies (Tokyo, Japan). OY-M *rrnB* promoter activity was assessed using the pET-P_*T7*_(RpoD) or pET-P_*T7*_(FliA) expression vectors, which carried OY-M *rpoD* or *fliA* genes controlled by the Isopropyl β-D-1-thiogalactopyranoside (IPTG)-induced T7 promoter, as well as the promoter-probe pACYC-P_*rrnB*_(GFP) or (Luc) vector, which carried a OY-M *rrnB* promoter fused to Δ*kan*::*gfp* or Δ*kan*::*luciferase* operon (Table 1 and Fig. 3). First, the full OY-M *rpoD* and *fliA* sequences were amplified by PCR using the primer sets described in Table S3 and the KOD DNA polymerase (TOYOBO, Shiga, Japan). Total DNA extracted from OY-M-infected plants, pGFP Vector (Takara), and pGL4 Luciferase Reporter Vector (Promega, Madison, WI) was used as PCR templates. The PCR product was digested with *Nde*I and *Xho*I restriction enzymes for *rpoD*, or *Nde*I and *Hin*dIII for *fliA*, and then cloned into the pET-30a vectors (Novagen, Madison, WI) through the same sites. Next, the promoter region of OY-M *rrnB* (300 bp upstream of the gene), *luciferase*, and *gfp* were separately amplified by PCR using the primer sets described in Table S3 and KOD DNA polymerase. The amplified fragments of the *rrnB* promoter, *luciferase*, and *gfp* were used as templates for recombinant PCR. The *rrnB* promoter fused to the *luciferase* or *gfp* fragments was amplified by recombinant PCR by annealing two complementary oligonucleotides, respectively, that were designed to contain *Hin*dIII restriction sites near the 5′ end and *Xho*I sites near the 3′ end (Table S3). The annealed double-stranded DNA was inserted into the pACYC177 vector (Fermentas) at the *Hin*dIII and *Xho*I restriction sites.

**Table:** Plasmids used in this study

Plasmid	Description	Source
pET-30a	A expression vector with *lac*-inducible T7 promoter; ColE1; Kan^r^	Novagen
pET-RpoD	pET-30a carrying a OY phytoplasmal *rpoD* (*PAM628*); Kan^r^	This study
pET-FliA	pET-30a carrying a OY phytoplasmal *fliA* (*PAM320*); Kan^r^	This study
pACYC177	A low-copy-number plasmid; p15A; Amp^r^; Kan^r^	Fermentas
pACYC-P_*rrnB*_ (GFP)	A promoter-probe vector containing Δ*kan*::*gfp* with promoter region of OY phytoplasmal *rrnB* (PAM_r006) promoter; Amp^r^	This study
pACYC-P_*rrnB*_ (Luc)	A promoter-probe vector containing Δ*kan*::*luciferase* with promoter region of OY phytoplasmal *rrnB* (PAM_r006) promoter; Amp^r^	This study
pACYC-P_*rpsJ*_ (Luc)	A promoter-probe vector containing Δ*kan*::*luciferase* with promoter region of OY phytoplasmal *rpsJ* (*PAM199*) promoter; Amp^r^	This study
pACYC-P_*gyrB*_ (Luc)	A promoter-probe vector containing Δ*kan*::*luciferase* with promoter region of OY phytoplasmal *gyrB* (*PAM498*) promoter; Amp^r^	This study
pACYC-P_*289*_ (Luc)	A promoter-probe vector containing Δ*kan*::*luciferase* with promoter region of OY phytoplasmal *PAM289* promoter; Amp^r^	This study
pACYC-P_*mdlB*_ (Luc)	A promoter-probe vector containing Δ*kan*::*luciferase* with promoter region of OY phytoplasmal *mdlB* (*PAM059*) promoter; Amp^r^	This study
pACYC-P_*tengu*_ (Luc)	A promoter-probe vector containing Δ*kan*::*luciferase* with promoter region of OY phytoplasmal *tengu* (*PAM765*) promoter; Amp^r^	This study
pACYC-P_*hflB*_ (Luc)	A promoter-probe vector containing Δ*kan*::*luciferase* with promoter region of OY phytoplasmal *hflB* (*PAM064*) promoter; Amp^r^	This study
pACYC-P_*himA*_ (Luc)	A promoter-probe vector containing Δ*kan*::*luciferase* with promoter region of OY phytoplasmal *himA* (*PAM317*) promoter; Amp^r^	This study
pACYC-P_*dam*_ (Luc)	A promoter-probe vector containing Δ*kan*::*luciferase* with promoter region of OY phytoplasmal *dam* (*PAM565*) promoter; Amp^r^	This study

Promoter activities of other OY-M genes in *E. coli* were assessed using the pET-P_*T7*_(RpoD) or pET-P_*T7*_(FliA) expression vectors with the pACYC-P_OY_(Luc) promoter-probe vector carrying the selected OY-M gene promoter fused to the Δ*kan*::*luciferase* operon (Table 1). The promoter-containing regions located 300 bp upstream of the OY-M *rpsJ, gyrB, PAM289, mdlB, tengu, hflB, himA,* and *dam* genes (P_*rpsJ*_, P_*gyrB*_, P_*289*_, P_*mdlB*_, P_*tengu*_, P_*hflB*_, P_*himA*_ and P_*dam*_, respectively) were separately amplified by the primer sets described in Table S3 that were designed to incorporate *Hin*dIII restriction sites near the 5′ end and *Nde*I sites near the 3′ end. The annealed double-stranded DNAs were inserted into the pACYC-P_*rrnB*_(Luc) at the *Hin*dIII and *Nde*I restriction sites (Fig. 3). The resulting constructs contained the various specified promoters upstream of the *luciferase* reporter genes (Table 1).

### Determination of promoter activity by GFP imaging

Promoter activity was determined by the presence of green fluorescent protein (GFP) fluorescence. *Escherichia coli* BL21-CodonPlus (DE3)-RIL cells (Stratagene, La Jolla, CA) were transformed with pACYC-P_*rrnB*_(GFP) and either pET-P_*T7*_(RpoD) or pET-P_*T7*_(FliA). *Escherichia coli* cells transformed with pACYC-P_*T7*_(GFP) (encoding T7 promoter upstream of *gfp*) or pACYC-P_*rrnB*_(GFP) alone were used as positive and negative controls, respectively. *Escherichia coli* cells harboring the appropriate plasmids as well as Amp^r^ and Kan^r^ antibiotic resistance genes were grown overnight in LB medium supplemented with ampicillin (50 μg/mL) and kanamycin (20 μg/mL) at 37°C. Cultures were diluted 1:100 in fresh LB medium to an optical density at 600 nm (OD_600_) of 0.4–0.8. Note that *E. coli* cells in OD = 0.4–0.8 are usually in the exponential growth phase. RpoD or FliA expression was induced by adding IPTG to the cultures at a final concentration of 0.1 mmol/L. After the induction by adding IPTG, cells were grown for 1.5 h. To observe the GFP fluorescence intensity expressed in *E. coli*, 10-μL aliquots of cultured cells were placed in glass slides and analyzed using an Axio Imager Z1 microscope (Carl Zeiss MicroImaging GmbH, Jena, Germany). An AxioCam HRc camera (Carl Zeiss MicroImaging GmbH) used to collect images was controlled by AxioVision Release 4.6 software (Carl Zeiss MicroImaging GmbH).

### Determination of promoter activity by luciferase assay

Promoter activity was quantified by measuring luciferase activity. *Escherichia coli* BL21-CodonPlus (DE3)-RIL cell (Stratagene) was transformed with pACYC-P_*rrnB*_(Luc) and either pET-P_*T7*_(RpoD) or pET-P_*T7*_(FliA). The cells harboring the two types of appropriate plasmids were grown and RpoD and FliA was induced as described above. To determine the promoter activity of the OY-M *rrnB, rpsJ, gyrB, PAM289, mdlB, tengu, hflB, himA,* and *dam* genes induced by RpoD or FliA, cultured cells were collected at different time points (0, 0.5, 1, and 1.5 h) and cell densities were determined by OD_600_ measurement. A commercial luciferase assay system (Promega) was used in this study, as follows. Ten microliters of buffer consisting of 1 mol/L K_2_HPO_4_ (pH 7.8) and 20 mmol/L EDTA was added to 90-μL aliquots of cultured cells. The mixtures were snap frozen on dry ice and brought to room temperature. Lysates were prepared by resuspending the thawed *E. coli* cell suspension in 300 μL of cell culture lysis buffer containing 25 mmol/L Tris-phosphate (pH 7.8), 2 mmol/L dithiothreitol, 2 mmol/L 1,2-diaminocyclohexane-*N, N, N’, N’*-tetraacetic acid, 10% glycerol, 1% Triton X-100, 0.125% lysozyme, and 0.25% bovine serum albumin. The cell lysates were thoroughly mixed by vortexing for 1 min. Twenty microliters of the lysates was mixed with 100 μL of luciferase assay reagent. Luciferase activity was measured for 10 sec using a BLR-201 Luminescence reader (Aloka, Tokyo, Japan). The luciferase activity measurements by individual OY-M promoters were repeated a total of three times. Measurements are reported as relative luciferase units (RLU)/OD_600_. Results are expressed as the mean ± SE. Significant differences between the mean values of the groups were evaluated by Student's *t*-test and with Statcel software (OMS Publishing).

### SDS-page

To confirm RpoD or FliA expression in *E. coli* cells, the expression of RpoD or FliA protein in *E. coli* was induced by IPTG treatment, and cultured cells were collected at different time points. The cell extracts were electrophoresed in a sodium dodecyl sulfate (SDS)-polyacrylamide gel. The polyacrylamide concentration for SDS-PAGE was 12.5% for the detection of RpoD expression and 15% for the detection of FliA expression. Signal intensity was quantified using Adobe Photoshop version 7.0 software (Adobe Systems Inc., Mountain View, CA) and ImageJ software (National Institutes of Health, Bethesda, MD).

## Results

### OY-M σ factors and RNAP expression levels during insect and plant host switching

**Figure 1:**
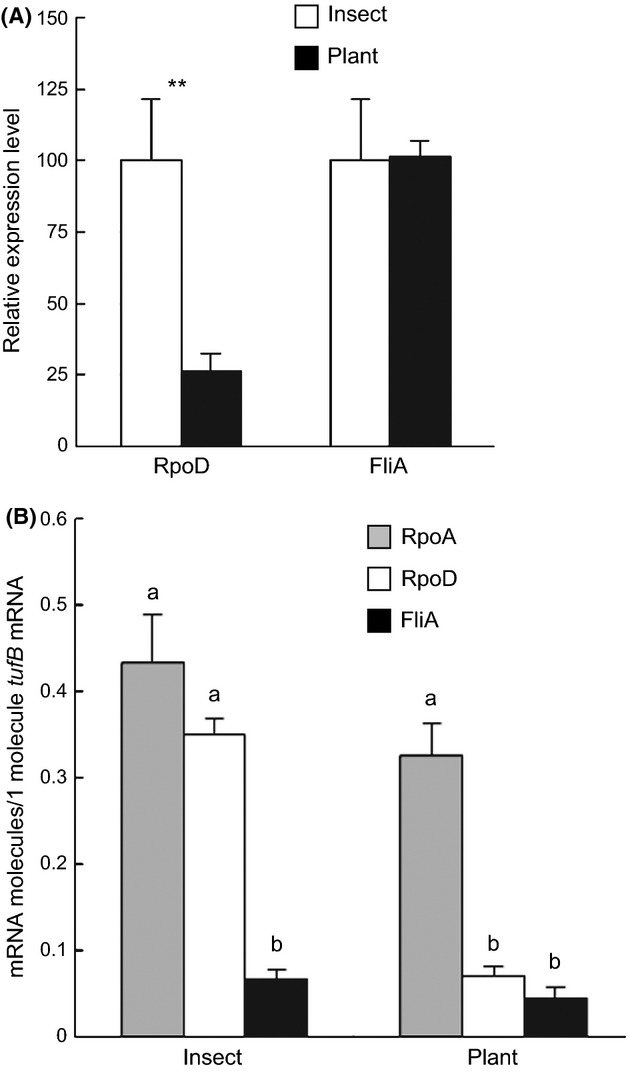
Transcriptional expression levels of OY-M-derived *rpoD, fliA,* and *rpoA* in insect and plant hosts. (A) Relative quantification of *rpoD* and *fliA*mRNAs in insect and plant hosts. Transcription of *rpoD* and *fliA*mRNAs was examined by qRT-PCR and the results were normalized against *tufB* expression. The expression level of the σ factors in insect host were adjusted to 100%. Error bars indicate SE. **A significant difference of *P *<* *0.01 (Student's *t*-test). Note that differences in the expression level between RpoD and FliA could not be compared because the quantitative values are relative. (B) Absolute quantification of *rpoA, rpoD,* and *fliA*mRNAs. The numbers of *rpoA, rpoD,* and *fliA*mRNA molecules were examined by absolute qRT-PCR and the results were normalized against the number of *tufB*mRNA molecules. Error bars indicate SE. “a” and “b” are significantly different at the 5% level by Scheffe's test. Note that all six expression levels are compared.

To determine whether the σ factors of OY-M phytoplasmas were differentially expressed in infected insect or plant hosts, the relative mRNA transcriptional levels of the *rpoD* and *fliA* OY-M σ factors were measured by qRT-PCR using *tufB* as an internal control. As a result, the mRNA of *rpoD* was approximately four times more abundant in OY-M-infected insects than in OY-M-infected plants (*P *<* *0.01), while *fliA* mRNA expression did not differ significantly between the hosts (Fig. 1A).

To further investigate differences in expression, absolute copy number was quantified by qRT-PCR using pure RNA standards of *tufB, rpoA, rpoD,* and *fliA*, where *rpoA, rpoD*, and *fliA* mRNA molecules were compared to one molecule of *tufB* mRNA of phytoplasmas in both insect and plant hosts. Similar to the results obtained from the relative mRNA expression experiment, *rpoD* mRNA molecules were more abundant in insect than in plant hosts, and the numbers of *fliA* mRNA molecules were almost equal between these hosts (Fig. 1B). The *rpoA* mRNA encoding the α subunit of the core RNAP enzyme was also constant between insect and plant hosts (Fig. 1B). In insect hosts, *fliA* mRNA expression was significantly lower (*P *<* *0.05) than both *rpoA* and *rpoD* mRNA expressions, which were approximately equal to each other (Fig. 1B). In plant hosts, *rpoA* mRNA expression was significantly higher than both *rpoD* and *fliA* mRNA expressions, and there was no significant difference between *rpoD* and *fliA* mRNA expressions (*P *<* *0.05) (Fig. 1B).

### OY-M gene expression levels during insect and plant host switching

**Figure 2:**
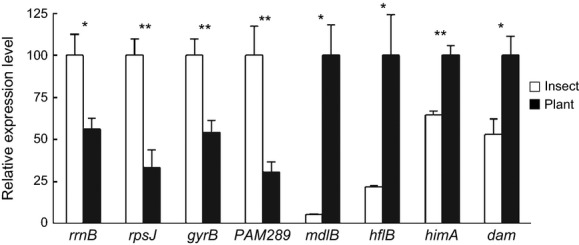
Transcriptional expression levels of eight OY-M genes in insect and plant hosts. Relative quantification of *rrnB, rpsJ, gyrB, PAM289, mdlB, hflB, himA,* and *dam*mRNA expression in insect and plant hosts was performed by qRT-PCR and the results were normalized against *tufB*. The *PAM289* data graph was redrawn based on previous data (Oshima et al. 2011). The *rrnB, rpsJ, gyrB,* and *PAM289* gene expression levels in insect hosts and the *mdlB, hflB, himA,* and *dam* gene expression levels in plant hosts were adjusted as 100%. Error bars indicate SE. * and **Significant differences of *P *<* *0.05 and *P *<* *0.01, respectively (Student's *t*-test).

Eight OY-M genes, including *rrnB, rpsJ, gyrB, PAM289, mdlB, hflB, himA,* and *dam*, were selected as representative σ-factor-regulated genes based on the microarray data of OY-M in the host switching between insect and plant hosts (Oshima et al. 2011). We measured the relative expression levels of these genes in insect and plant hosts by qRT-PCR using *tufB* as an internal control. The expression of four genes, *rrnB* (16S ribosomal RNA), *rpsJ* (30S ribosomal subunit protein S10 gene), *gyrB* (β subunit of DNA gyrase), and *PAM289* (unknown membrane protein), was significantly upregulated in infected insects compared to plants (Fig. 2), where *rrnB, rpsJ,* and *gyrB* were thought to be OY-M housekeeping genes. The expression of the other four genes, *mdlB* (ATP-binding component of the ABC-type multidrug transporter), *hflB* (ATP-dependent zinc protease), *himA* (one of two subunits of histone-like protein), and *dam* (DNA methyltransferase), was significantly upregulated in infected plants compared to insects (Fig. 2).

### Establishment of the *E. coli*-based ex vivo reporter assay system to evaluate the interaction between OY-M σ factors and phytoplasmal gene promoters

**Figure 3:**
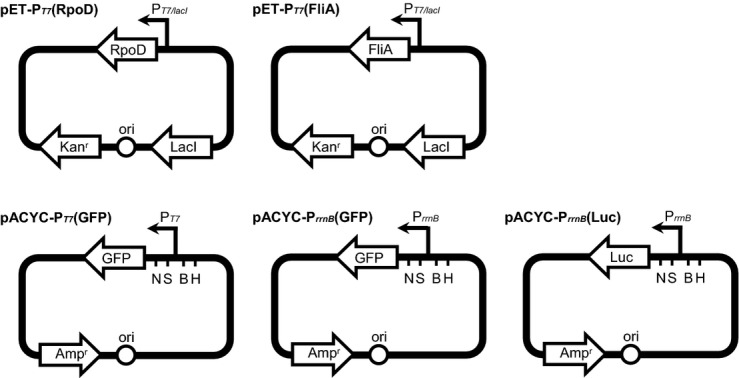
Schematic representation of the pET-P_*T7*_(RpoD), pET-P_*T7*_(FliA), pACYC-P_*T7*_(GFP), pACYC-P_*rrnB*_(GFP), and pACYC-P_*rrnB*_(Luc) plasmids. Ori, *Escherichia coli* replication origin; Kan^r^ and Amp^r^, genes conferring resistance to kanamycin and ampicillin, respectively; RpoD, OY-M *rpoD* gene without its own promoter; FliA, OY-M *fliA* gene without its own promoter; *Lac*I, lactose repressor; P_T7/lacI_, T7 promoter regulated by *Lac*I; P_*rrnB*_, transcription promoter of the OY-M *rrnB* gene; GFP,*gfp* reporter gene; Luc, *luciferase* reporter gene; N, *Nde*I; S, *Sal*I; B, *Bgl*II; H, *Hin*dIII.

**Figure 4:**
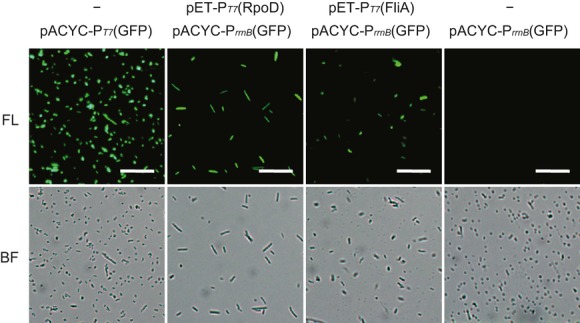
The *Escherichia coli*-based ex vivo reporter assay system using green fluorescent protein (GFP). Plasmids introduced into *E. coli* cells are shown above the relevant images. After P_T7_ promoter induction by Isopropyl β-D-1-thiogalactopyranoside (IPTG) treatment, GFP fluorescence was examined in each *E. coli* cell by fluorescence microscopy. *E. coli* cells containing pACYC-P_T7_(GFP) or pACYC-P_*rrnB*_(GFP) alone were used as positive or negative controls, respectively (left and right panels). FL, fluorescence; BF, bright field. Scale bar = 25 μm. GFP fluorescence was observed in *E. coli* containing pET-P_*T7*_(RpoD) plus pACYC-P_*rrnB*_(GFP) and pET-P_*T7*_(FliA) plus pACYC-P_*rrnB*_(GFP) (middle panels), suggesting that this system was successful in assaying for σ factor expression.

To study the mechanism regulating the observed differential σ factor expression, we needed to overcome the experimental obstacle that phytoplasmas cannot be cultured in vitro. Therefore, we developed an EcERA system that monitored the binding activity between phytoplasmal promoters and the RpoD or FliA σ factors. To analyze RpoD and FliA functions and to identify the genes regulated by these σ factors in the OY-M genome, we first constructed four plasmids: pET-P_*T7*_(RpoD) and pET-P_*T7*_(FliA), which carry the T7 promoter upstream of *rpoD* and *fliA*, respectively, as well as pACYC-P_*rrnB*_(GFP) and pACYC-P_*T7*_(GFP), which carry the OY-M *rrnB* promoter (used as a representative promoter downstream of σ factor expression) and the IPTG-activated T7 promoter upstream of *gfp,* respectively (Table 1 and Fig. 3). Four *E. coli* transformants were prepared for the reporter assay as shown in Figure 4. Following the induction of T7 polymerase by IPTG treatment, significant GFP fluorescence was observed in the positive-control pACYC-P_*T7*_(GFP)-transformants, but not in the negative-control pACYC-P_*rrnB*_(GFP)-transformants (Fig. 4; left and right images). These results suggest that neither the T7 polymerase nor the inherent *E. coli* σ factors recognized the promoter region of the OY-M *rrnB* gene. In contrast, GFP fluorescence was observed in both *E. coli* transformants cotransfected with pACYC-P_*rrnB*_(GFP) and either pET-P_*T7*_(RpoD) or pET-P_*T7*_(FliA) after IPTG treatment (Fig. 4; middle images). These results indicate that the RpoD and FliA σ factors can bind to *E. coli* RNA polymerase (RNAP_EC_) and be functional as RNAP_EC_-RpoD and RNAP_EC_-FliA holoenzymes to initiate OY-M *rrnB* promoter transcription. Taken together, these results suggest that the EcERA system is an effective tool to analyze RpoD and FliA regulation of phytoplasmal promoters.

### Evaluation of RpoD or FliA promoter-specific transcription by EcERA

**Figure 5:**
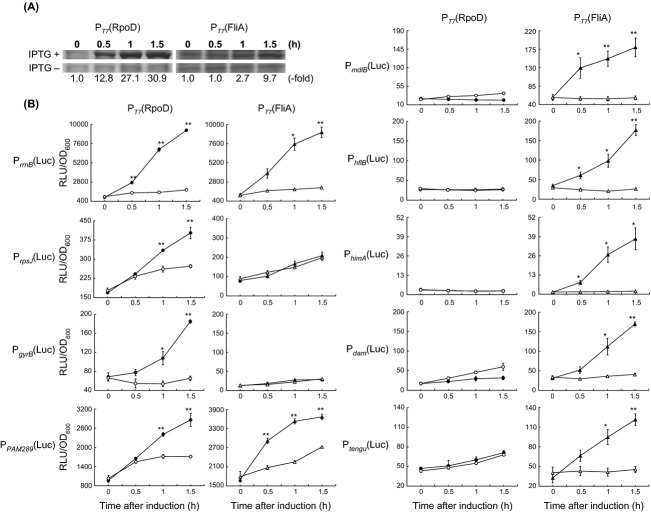
The *Escherichia coli*-based ex vivo reporter assay system using luciferase. (A) RpoD and FliA expression levels. RpoD and FliA protein levels in *E. coli* cells were analyzed by SDS-PAGE with or without IPTG treatment. Protein extracted from equivalent cell numbers were loaded onto each lane representing each experimental time point (0–1.5 h). Below each lane, the “-fold” numbers represent protein band density from each IPTG+ lane subtracted by the density calculated from the respective IPTG- lane, and then normalized to the density value at 0 h. (B) Luciferase activity measurements of σ factors regulating different promoters. Relative light unit (RLU) values indicate the magnitude of luciferase activity. RLU readings were normalized according to cell density (OD_600_). Closed and open symbols (circle and triangle) indicate treatment with or without IPTG, respectively. Error bars indicate SE. * and **Statistically significant differences at *P *<* *0.05 and *P *<* *0.01, respectively (Student's *t*-test).

Establishment of the EcERA system allowed us to determine how specific σ factors regulated the expression of other OY-M genes. To quantify the ability of RpoD or FliA to bind to phytoplasmal promoters, we used *luciferase* as the reporter gene instead of *gfp* because luciferase fluorescence quantification is more accurate than GFP fluorescence quantification (Vesuna et al. 2005). To ensure that the luciferase works similar to GFP in this system, two *E. coli* transformants, namely, *E. coli* cotransfected with pACYC-P_*rrnB*_(Luc) and either pET-P_*T7*_(RpoD) or pET-P_*T7*_(FliA), were prepared for the reporter assay as well as to monitor RpoD and FliA binding to the *rrnB* promoter, respectively. Indeed, gradually increasing RpoD and FliA protein expression was observed in IPTG-treated cells by SDS-PAGE analysis, but not in untreated control cells (Fig. 5A). Luciferase activity measured by fluorescence in both the RpoD and FliA transformants was gradually increased after IPTG treatment, but not in untreated control cells (Fig. 5B, P_*rrnB*_(Luc) panels). Significant differences were also observed between IPTG-treated and untreated cells, suggesting that the EcERA luciferase system is highly reproducible and reliably quantitative; thus, it is useful to analyze the binding function between exogenous σ factors and promoters.

Next, we exchanged the *rrnB* promoter sequence in the pACYC-P_*rrnB*_(Luc) plasmid with the promoter sequences from eight other OY-M genes to test whether they are also regulated by RpoD and/or FliA using the luciferase assay. These eight genes were as follows: three genes (*rpsJ, gyrB,* and *PAM289*) and four genes (*mdlB, hflB, himA,* and *dam*) were identified above to be highly expressed in insect and plant hosts, respectively (Fig. 2), and the *tengu* gene encoded a virulence factor that was previously reported to be highly expressed in plant hosts (Hoshi et al. 2009). While the luciferase activity was increased by RpoD expression in the *E. coli* transformants containing the *rrnB, rpsJ, gyrB,* and *PAM289* promoters (Fig. 5B), the luciferase activity was increased by FliA expression in the *E. coli* transformants containing the *rrnB, PAM289, mdlB, tengu, hflB, himA,* and *dam* promoters (Fig. 5B). These results suggest that the highly expressed genes in insect hosts were mainly regulated by RpoD, or both RpoD and FliA, and that the highly expressed genes in plant hosts were mainly regulated by FliA.

## Discussion

In this study, we established the novel “EcERA system” using the model bacterium *E. coli* to analyze the interaction between σ factors and promoters from unculturable bacteria, phytoplasma. This system was successfully established by “fine-matching” between *E. coli* RNA polymerase (RNAP_EC_) and two σ factors from phytoplasma, RpoD and FliA. While very little is known about gene regulatory systems in phytoplasmas because culturing phytoplasmas in vitro has not yet been achieved (Weintraub and Beanland 2006), our study revealed that RpoD and FliA are key transcriptional factors of phytoplasma during the host switching between insects and plants.

Here, we observed that *rpoD* mRNAs were more highly expressed in insect than in plant hosts (Fig. 1A), and that RNAP_EC_-RpoD-mediated RpoD expression initiated transcription from the *rrnB, rpsJ, gyrB,* and *PAM289* promoters that were also highly expressed in insect hosts (Fig. 5B). These results suggest that the RpoD σ factor regulates genes required for the OY-M response to insect hosts. In many bacteria, RpoD (σ^70^ factor) is responsible for recognizing the promoter regions of housekeeping genes in exponentially growing cells and is essential for cell survival (Ishihama 2000). Similarly, OY-M RpoD-regulated genes essential for cell survival, such as those related to translation (*rrnB, rpsJ*) and DNA replication (*gyrB*) (Hutchison et al. 1999; Kobayashi et al. 2003). Because the *rpsJ* gene is located at the most 5′ end of the S10-*spc* ribosomal protein gene operon (Miyata et al. 2002), and most genes encoded on this operon were significantly upregulated in insect hosts as compared to plant hosts (Oshima et al. 2011), OY-M RpoD likely regulates most ribosomal proteins. This result further suggests that the biological activity of OY-M, including protein synthesis, cell division, and cell growth, might be enhanced in insect hosts compared to plant hosts. In contrast to RpoD, *fliA* mRNA expression was similar in both hosts (Fig. 1A), even though the FliA-induced gene expressions of *mdlB, tengu, hflB, himA,* and *dam* were higher in plant hosts (Fig. 5B). Because phytoplasma is thought to differentially express transporter genes (such as *mdlB*) and secreted proteins (such as *tengu*) for the response to each host (Oshima et al. 2011), these results suggest that FliA primarily regulates genes required for OY-M response to plant hosts. Taken together, the results suggest that RpoD and FliA play central roles in the ability of OY-M to dramatically change its gene expression in the host switching between the insect and plant hosts. The Lyme disease agent, *Borrelia burgdorferi*, colonizes both a mammalian host and an arthropod vector host during its infectious cycle (Stanek and Strle 2003). One of the two alternative σ factors in *B. burgdorferi*, σ^54^, has been shown to be required for achieving mammalian infection and vector transmission (Fisher et al. 2005). Therefore, our data presented here support the idea that bacterial σ factors, such as those in phytoplasma and *B. burgdorferi,* play crucial roles in regulating gene expression for host switching.

While FliA expression, which regulated the host switching genes in plants, was constant between the insect and plant hosts (Figs. 1A, 5B), *rpoD* mRNA expression was significantly downregulated in plant hosts (Fig. 1B). Meanwhile, the expression of the nonsigma factor *rpoA*, encoding the α subunit within the RNAP core enzyme complex (α_2_ββ′γ), was constant between insect and plant hosts (Fig. 1B). The simplest way to explain these results is that σ factors compete for the limited binding capacity of the RNAP core enzyme, and RpoD downregulation allows FliA to bind to RNAP in plant hosts (Fig. S3). Indeed, it has been previously shown that if the available RNAP core protein is limited in a cell, decreasing the number of σ factors by one can actually induce genes that require another σ factor by allowing that factor to bind to the RNAP core protein (Farewell et al. 1998). This σ factor competition is extensively studied in *E. coli*, where gene expression is dramatically altered throughout the transition from the exponential growth phase to the stationary phase (Jishage and Ishihama 1995; Jishage et al. 1996). *Escherichia coli* gene expression in the exponential growth and stationary phases is mainly regulated by RpoD and RpoS (one of the alternative σ subunits), respectively (Jishage and Ishihama 1995; Jishage et al. 1996), both of which compete for limited amount of RNAP (Farewell et al. 1998; Jishage et al. 2002). While RpoD and RNAP core enzyme are constitutively expressed in both the exponential growth and the stationary phase (Farewell et al. 1998; Jishage et al. 2002), RpoS has a dynamic expression pattern where its expressional level is extremely low in the exponential growth phase but is markedly increased upon the entry into the stationary phase (Jishage et al. 2002). As a result of the increase of RpoS, the genes required for the stationary phase are upregulated by RpoS while RpoD-regulated genes are downregulated because of the limited amount of RNAP core enzyme (Farewell et al. 1998; Jishage et al. 2002). Several other factors in *E. coli*, such as the anti-sigma factor Rsd or 6S RNA, could also facilitate the transcription switchover during starvation by inhibiting RpoD-driven transcription (Jishage and Ishihama 1998; Wassarman and Storz 2000; Trotochaud and Wassarman 2005). However, no genes encoding proteins homologous to these anti-sigma factors are found in the OY-M phytoplasma genome (Oshima et al. 2004); thus, other mechanisms may control OY-M FliA expression.

The genes *rrnB* and *PAM289* were highly expressed in insect hosts, and our EcERA assay results indicate that they were regulated by both RpoD and FliA (Fig. 5B). The higher expression levels of both of *rrnB* and *PAM289* in insect hosts might be explained by the abundant RpoD in insect hosts, where RpoD-induced gene expression in insect hosts might be higher than the gene expression induced by both RpoD and FliA in plant hosts (Fig. 1B and S3).

Bacteria frequently use two-component signal transduction regulatory systems to sense the environmental changes (Robinson et al. 2000). These two-component systems usually are composed of a membrane-associated histidine kinase, the sensor, and a response regulator, which acts in the cytoplasm. The sensor detects the environmental signal or stress, and the regulatory protein triggers the cellular response via gene transcription modulation by transcription factors, including sigma factors. The gene expression of OY-M RpoD was sufficiently changed upon the host switching between insect or plant hosts. However, the two-component systems are not encoded within the OY-M genome (Oshima et al. 2004). Phytoplasma might govern the response to insect and plant hosts by an unknown environmental response system.

Phytoplasma genomes contain many multicopy gene clusters called PMUs that are thought to be prophage sequences originating from phage attacks (Wei et al. 2008) and encode multiple redundant genes related to DNA replication (*ssb, dnaB*, and *dnaG*), nucleotide synthesis (*tmk*), recombination (*himA*); membrane-bound and secreted proteins; and unknown proteins (Bai et al. 2006; Arashida et al. 2008; Kube et al. 2008; Tran-Nguyen et al. 2008). As noted earlier, *fliA* genes are also encoded within a PMU region (Arashida et al. 2008). Here, we showed that FliA regulated several PMU genes, including *hflB, himA,* and *dam* (Fig. 5B). Previous studies showed that acquired DNA sequences benefit a recipient bacterium only if they are expressed at the right time, in the correct location, and in a coordinated manner (Perez and Groisman 2009). Therefore, a foreign DNA segment usually includes a regulatory gene element that accomplishes these expression patterns. For example, the SPI-2 pathogenicity island of *Salmonella enterica* harbors a large number of structural genes that are coordinately regulated by the SsrB/SpiR two-component system, which is encoded within the SPI-2 locus (Fass and Groisman 2009). The enterohemorrhagic *E. coli* genome contains the LEE pathogenicity island essential for full virulence that is regulated by many regulatory factors, including Ler, GrlA, and GrlR, which are also encoded within the LEE locus (Mellies et al. 2007). In phytoplasma, PMUs were reported to contribute to host adaptation (Toruño et al. 2010); therefore, FliA can be considered a regulatory factor that regulates itself as well as functions to govern transcriptional switching during adaptation to insect and plant hosts.

In this study, we demonstrated that the *E. coli*-derived RNAP holoenzymes containing the phytoplasma σ factors, RpoD and FliA, were able to initiate transcription from phytoplasma-derived promoters. *Escherichia coli* was chosen in part because it encodes seven σ factors, including RpoD and FliA (Ishihama 2000). The overall amino acid similarity and identity between OY-M- and *E. coli*-derived RpoD is 41% and 26%, respectively, and 33% and 14% between OY-M- and *E. coli*-derived FliA, respectively (Figs. S1, S2, and Table S4). Sigma (σ) factors usually have four sequence motifs (subregions) related to RNAP binding and promoter sequence recognition (Borukhov and Severinov 2002). Because subregions 2.1 and 2.2 are implicated in core binding (Murakami et al. 2002a,b), the ability of the OY-M σ factors to bind to and function with RNAP_EC_ could be explained by the highly conserved amino acid sequences in these two regions (Figs. S1, S2, and Table S4). *Chlamydia trachomatis* FliA also possesses highly conserved subregions 2.1 and 2.2 that mediate binding to RNAP_EC_ and induce transcriptional expression from a *C. trachomatis* FliA-dependent promoter (Shen et al. 2004). Subregions 2.4 and 4.2 are involved in promoter sequence recognition, and are also highly conserved between OY-M and *E. coli* (Figs. S1, S2). *Escherichia coli* cells expressing OY-M-derived RpoD or FliA exhibited abnormally long cell shapes and low cell densities (Fig. 4), which could be a consequence of this high conservation between the OY-M and *E. coli* σ factors, because OY-M RpoD and FliA could affect the *E. coli* gene expression system that alters cell shape and cell growth. In addition, the background signals in Figure 5B (lux activity in zero time point) were quite different between each promoter construct, suggesting that the endogenous *E. coli* sigma factors could recognize the phytoplasma promoters. However, the effect of the endogenous *E. coli* sigma factors on this EcERA system would be smaller than that of OY-M sigma factors because OY-M sigma factors were overexpressed by adding IPTG and could be visualized even by normal SDS-PAGE (Fig. 5A). Taken together, our data demonstrate that a series of transcriptional regulatory elements and mechanisms are highly conserved among phylogenetically distant bacteria.

We established here a novel analysis method using *E. coli* as a model system that allowed us to study the regulatory mechanisms of gene expression present in unculturable phytoplasma bacteria. This study reveals that phytoplasmal σ factors participate in the transcriptional regulation of a group of genes that are involved in the adaptation response to the different environments, that is, the insect and plant hosts. Previous studies showed that the proportion of unculturable bacteria within the vast natural bacterial species variety is extremely high (Amann et al. 1995); this was confirmed by more recent metagenomics analysis (Venter et al. 2004; Eisen 2007; Yooseph et al. 2007). The novel strategy to analyze promoter regulation and activity using hetero-RNAP-σ complexes developed here could be more broadly applied to discover unknown transcriptional expression regulation mechanisms in other unculturable bacteria in the future.

## Supporting Information

**Table S1.** In vitro transcription primers used in this study. **Table S2.** Quantitative Real-time RT-PCR primers used in this study. **Table S3.** Molecular cloning primers used in this study. **Table S4.** Amino acid sequence identity and similarity scores of RpoD and FliA between OY-M and other bacteria.

**Figure S1.** and are indicated by lines above the alignment. Subregions 2.1 and 2.2 were reported to be involved in core binding, and subregions 2.4 and 4.2 were reported to be involved in promoter recognition **Figure S2.** Amino acid alignment of FliA. OY-M FliA (OY-M), *Bacillus subtilis* σ^F^ (Bacillus), *Chlamydia trachomatis* σ^28^ (Chlamydia), and *Escherichia coli* FliA (Escherichia) were aligned using the CLUSTAL W program. Amino acid similarity is indicated by highlighting (black shadow, >80% identity; gray shadow, 60–80% identity); gaps are indicated by hyphens. Several motifs (subregions) conserved among the many RpoD genes were defined based on previous studies (Lonetto et al. **Figure S1.** Amino acid alignment of RpoD. OY-M RpoD (OY-M), *Bacillus subtilis* σ^A^ (Bacillus), *Chlamydia trachomatis* σ^70^ (Chlamydia), and *Escherichia coli* σ^70^ (Escherichia) were aligned using the CLUSTAL W program. Amino acid similarity is indicated by highlighting (black shadow, >80% identity; gray shadow, 60–80% identity); gaps are indicated by hyphens. Several motifs (subregions) conserved among the many RpoD genes were defined based on previous studies (Lonetto et al.) and are indicated by lines above the alignment. Subregions 2.1 and 2.2 were reported to be involved in core binding, and subregions 2.4 and 4.2 were reported to be involved in promoter recognition **Figure S3.** A model illustrating the RpoD and FliA regulatory network in the OY-M bacterial cell during host adaptation. RpoD is significantly more abundant than FliA in insect hosts. RpoD binding to RNAP regulates the *rrnB, rpsJ, gyrB,* and *PAM289* genes (filled arrows) that were highly expressed in insect hosts. RpoD and FliA exist in approximately equal amounts in plant hosts. FliA binding to RNAP regulates the *mdlB, tengu, hflB, himA,* and *dam* genes (open arrows) that were highly expressed in plant hosts.
