# Supplementary material for: New ex vivo reporter assay system reveals that σ factors of an unculturable pathogen control gene regulation involved in the host switching between insects and plants
Source: Microbiologyopen. 2013 May 31;2(4):553–65. doi: 10.1002/mbo3.93 (PMC3831623; doi:10.1002/mbo3.93)
Supplement: Supplementary file 1 — Table S1. In vitro transcription primers used in this study. Table S2. Quantitative Real-time RT-PCR primers used in this study. Table S3. Molecular cloning primers used in this study. Table S4. Amino acid sequence identity and similarity scores of RpoD and FliA between OY-M and other bacteria. [file mbo30002-0553-sd1.doc]

**Table S1.** *In vitro* transcription primers used in this study

| Primer name | Primer sequence |
| --- | --- |
| *rpoA*_iv (F) | CCGCTCGAGATGAAAAATATTAAATTTATTAAAC |
| *rpoA*_iv (R) | GGAATTCCTAATTGTGAGCTTCTTTTTTAGAT |
| *rpoD*_iv (F) | CCATCGATATGGAATTCGATAACATAATCAAAA |
| *rpoD*_iv (R) | CGGGATCCTTATTTGTGGTTGTGGTACAAACTT |
| *fliA*_iv (F) | CCATCGATATGTTAAGACAAAAATTATTTAAAG |
| *fliA*_iv (R) | CGGGATCCTTACGGACGAGAAAGGTTAACGTTT |
| *tufB_iv* (F) | CCATCGATATGGCTAATGAAAAATTTATACGAA |
| *tufB_iv* (R) | CGGGATCCTTAGTTAAGAAGTTTGGAAACTGAT |

**Table S2.** Quantitative Real-time RT-PCR primers used in this study

| Primer name | Primer sequence |
| --- | --- |
| *rrnB*_RT (F) | TGCTTAGGGAGGAGCTTGCG |
| *rrnB*_RT (R) | TCTCAGCCCGGCTACACATC |
| *rpsJ*_RT (F) | AGGTCCAATACCTCTTCCTACCAG |
| *rpsJ*_RT (R) | TGCGTCGTTCAAATTGTTCTC |
| *gyrB*_RT (F) | AAGCTTTAGCAGGACACGCTAAC |
| *gyrB*_RT (R) | TTCCTCTACCGTTGTCAGAAACAC |
| *PAM289*_RT (F) | TTTGAAAGTGCACCAAATGAC |
| *PAM289*_RT (R) | AAATCGTCTCCAATAGAAAGACC |
| *mdlB*_RT (F) | TGCTTCCAAAGTATCATCCTTGC |
| *mdlB*_RT (R) | TCTCAGTGGGCGAAAAAGTTTG |
| *hflB*_RT (F) | TCGATCAAGTCCGCCCTAAAG |
| *hflB*_RT (R) | AACATTAGCCTCACCACAAAGAGC |
| *himA*_RT (F) | AAGTAGTTTTATCACCTCAAATCGG |
| *himA*_RT (R) | TCTTTGTTTTAGGGTTTCTACCG |
| *dam*_RT (F) | GCGCTTTATATTTATCCTTAATGCC |
| *dam*_RT (R) | TTCCATATTTGGATGAGTTGTGTG |

**Table S3.** Molecular cloning primers used in this study

| Primer name | Primer sequence |
| --- | --- |
| RpoD (F) | GGAATTCCATATGGAATTCGATAAC |
| RpoD (R) | CCGCTCGAGTTATTTGTGGTTGTGG |
| FliA (F) | GGAATTCCATATGTTAAGACAAAAA |
| FliA (R) | CCCAAGCTTTTACGGACGAGAAAGG |
| GFP (F) | AATAATTTTGTTTAACTTTAAGAAGGAGATATACATATGAGTAAAGGAGAAGAACTTTTCA |
| GFP (R) | CCGCTCGAGTTATTTGTATAGTTCATCCATGCCA |
| Luc (F) | AATAATTTTGTTTAACTTTAAGAAGGAGATATACAATGGAAGACGCCAAAAACATAAAGA |
| Luc (R) | CCGCTCGAGTTACAATTTGGACTTTCCGCCCTTC |
| PT7 (F) | CCCAAGCTTAGATCTGCATGCAAGGAGATGGCGCCCAACA |
| PT7 (R) | TATATCTCCTTCTTAAAGTTAAACAAAATTATTCATATGGTCGACGGGGAATTGTTATCCGCTCACAATT |
| P*rrnB* (F) | CCCAAGCTTAGATCTATTTGCCGGAATTCAATCTTAAAAC |
| P*rrnB* (R) | TATATCTCCTTCTTAAAGTTAAACAAAATTATTCATATGGTCGACTTACAAAATTGATGCATCAAAAATT |
| P*rpsJ* (F) | CCCAAGCTTCTTATTGTATTTTTTAACAAACAAA |
| P*rpsJ* (R) | GGAATTCCATATGTTATATTCTCCTTTTTTAGAAATGT |
| P*gyrB* (F) | CCCAAGCTTTTTACTCAATAATTGTAAAAACCAC |
| P*gyrB* (R) | GGAATTCCATATGAAGAATAGCTCCTTTTTTGTAAATT |
| P*289* (F) | CCCAAGCTTGCATTTAAAAGAAGAAAAATATTTT |
| P*289* (R) | GGAATTCCATATGAAGTAATAATGCACTTAAACAATAA |
| P*mdlB* (F) | CCCAAGCTTAAGTCGATAAGAATATGTTACTTTT |
| P*mdlB* (R) | GGAATTCCATATGTAGTCTAAACTCACAATTCCTTTGT |
| P*tengu* (F) | CCCAAGCTTAAACCGTTTTAAAATGACCTGAATT |
| P*tengu* (R) | GGAATTCCATATGAATTTATATATCACCTGCTTTCTTT |
| P*hflB* (F) | CCCAAGCTTAACTAAAAACCCAACATCAACTAAA |
| P*hflB* (R) | GGAATTCCATATGTAAATTATTGACTAAGGCTTGTTGG |
| P*himA* (F) | CCCAAGCTTATCAACAATTAGCCTTGGAATTACA |
| P*himA* (R) | GGAATTCCATATGAATGATTTGACTCCTTTTTTTGATT |
| P*dam* (F) | CCCAAGCTTATCAACGAAGATTATCAACCTAGTT |
| P*dam* (R) | GGAATTCCATATGTATCCGAAATAAGGCTTATCGTTGT |

**Table S4** Amino acid sequence identity and similarity scores of RpoD and FliA between OY-M and other bacteria.

| **RpoD** | | | | | | |
| --- | --- | --- | --- | --- | --- | --- |
|  | **Overall sequence** | | | **Sub-regions 2.1 and 2.2** | | |
|  | ***Bacillus***  ***subtilis*** | ***Chlamydia trachomatis*** | ***E. coli*** | ***Bacillus***  ***subtilis*** | ***Chlamydia trachomatis*** | ***E. coli*** |
| Identity (%) | 36 | 27 | 26 | 75 | 71 | 71 |
| Similarity (%) | 49 | 43 | 41 | 88 | 84 | 86 |
| **FliA** | | | | | | |
|  | **Overall sequence** | | | **Sub-regions 2.1 and 2.2** | | |
|  | ***Bacillus***  ***subtilis*** | ***Chlamydia trachomatis*** | ***E. coli*** | ***Bacillus***  ***subtilis*** | ***Chlamydia trachomatis*** | ***E. coli*** |
| Identity (%) | 17 | 19 | 14 | 34 | 40 | 23 |
| Similarity (%) | 35 | 38 | 33 | 60 | 63 | 55 |
