# Supplementary figures and images for: New ex vivo reporter assay system reveals that σ factors of an unculturable pathogen control gene regulation involved in the host switching between insects and plants
Source: Microbiologyopen. 2013 May 31;2(4):553–65. doi: 10.1002/mbo3.93 (PMC3831623; doi:10.1002/mbo3.93)

# Supplementary Figure S1

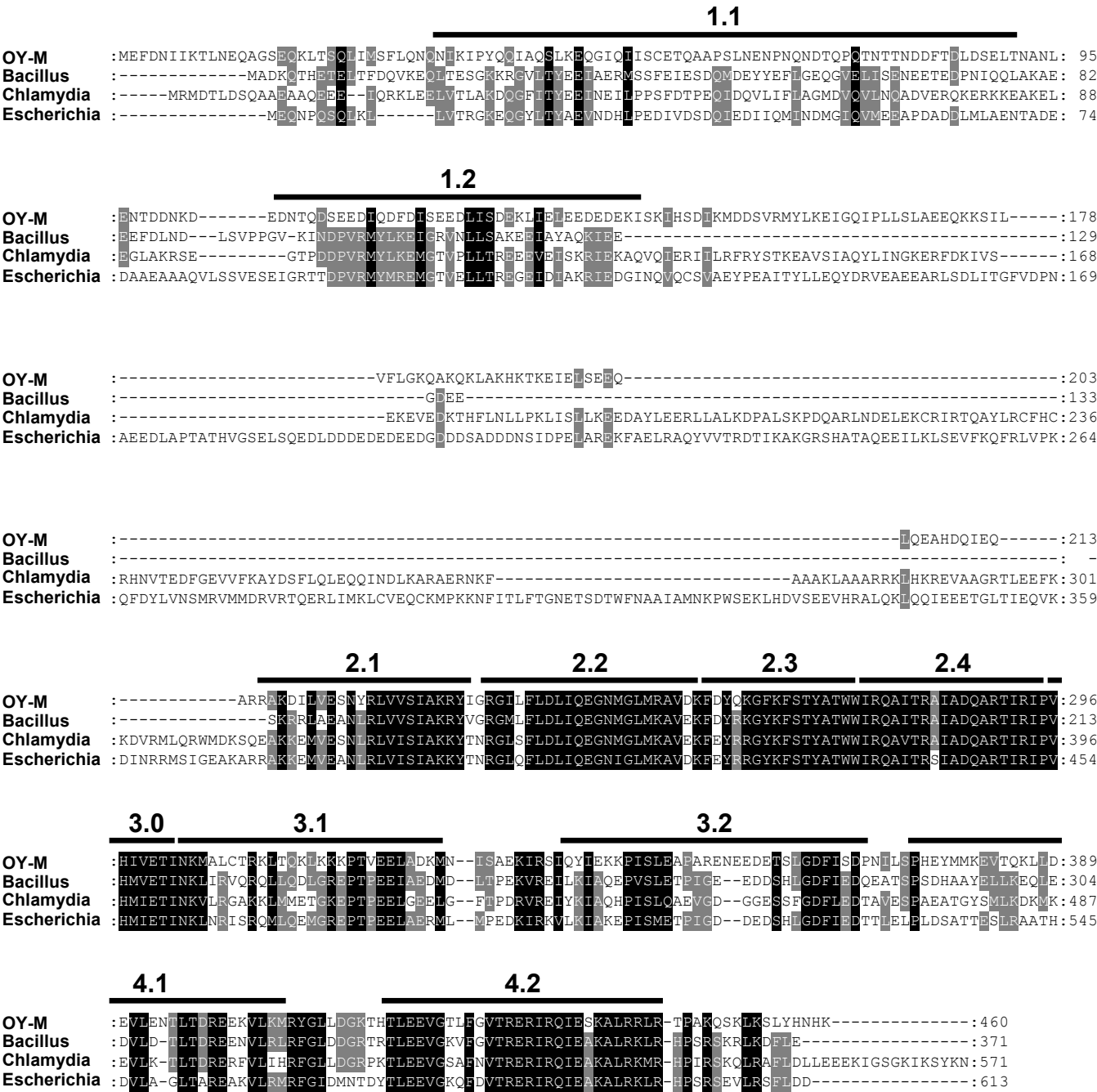

# Supplementary Figure S2

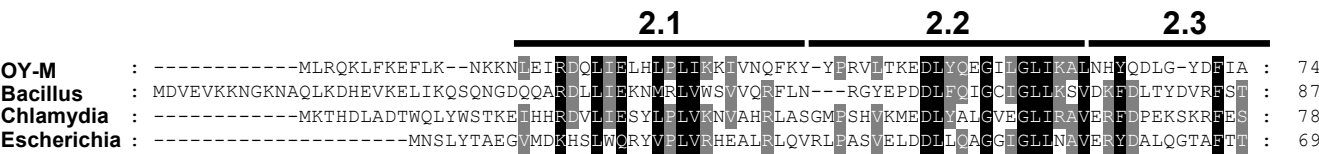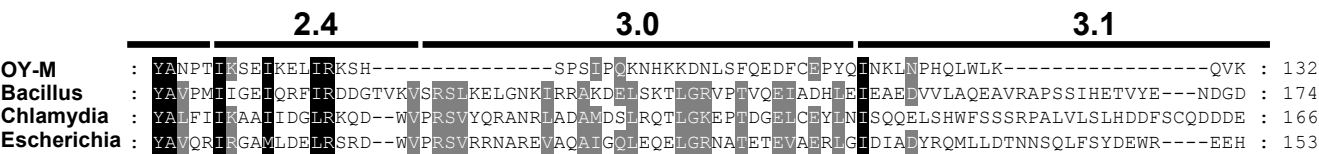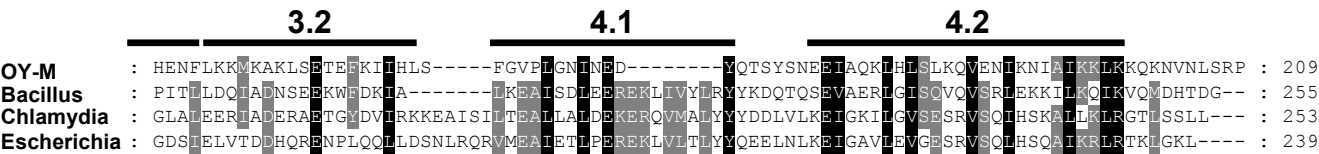

## Supplementary Figure S3

## Insect host

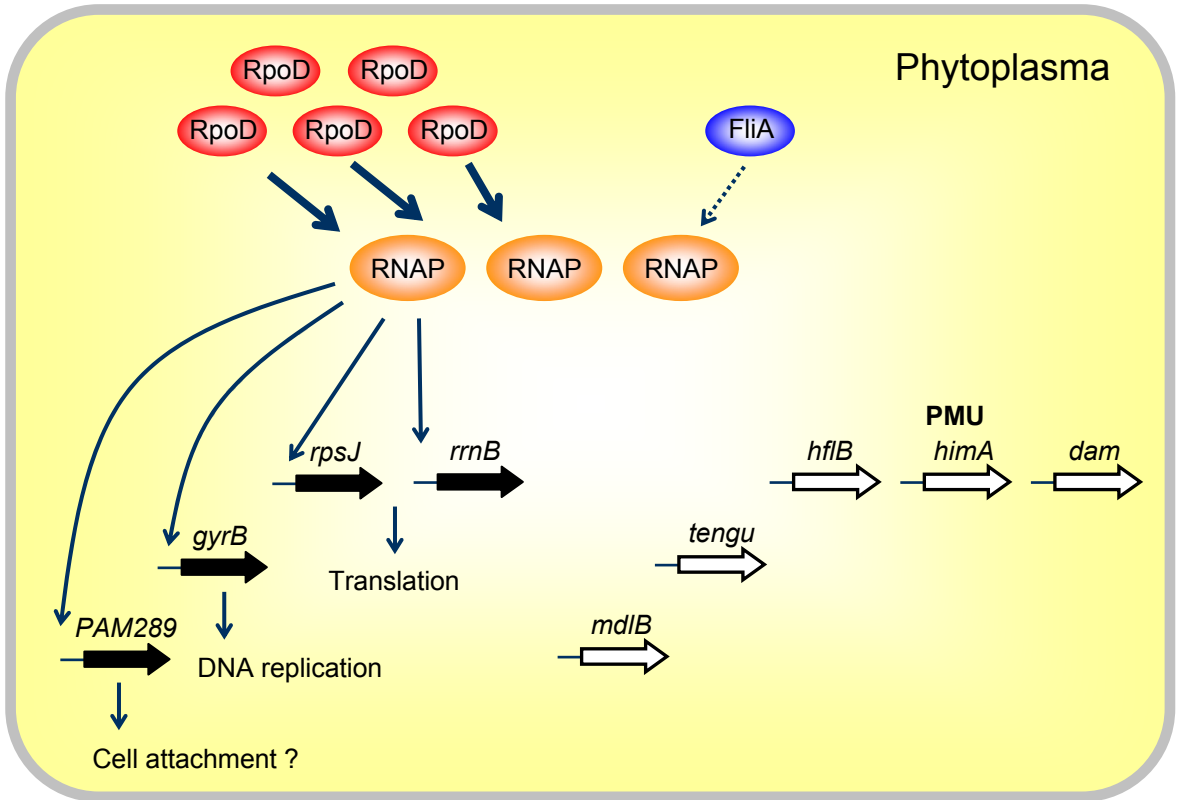

## Plant host

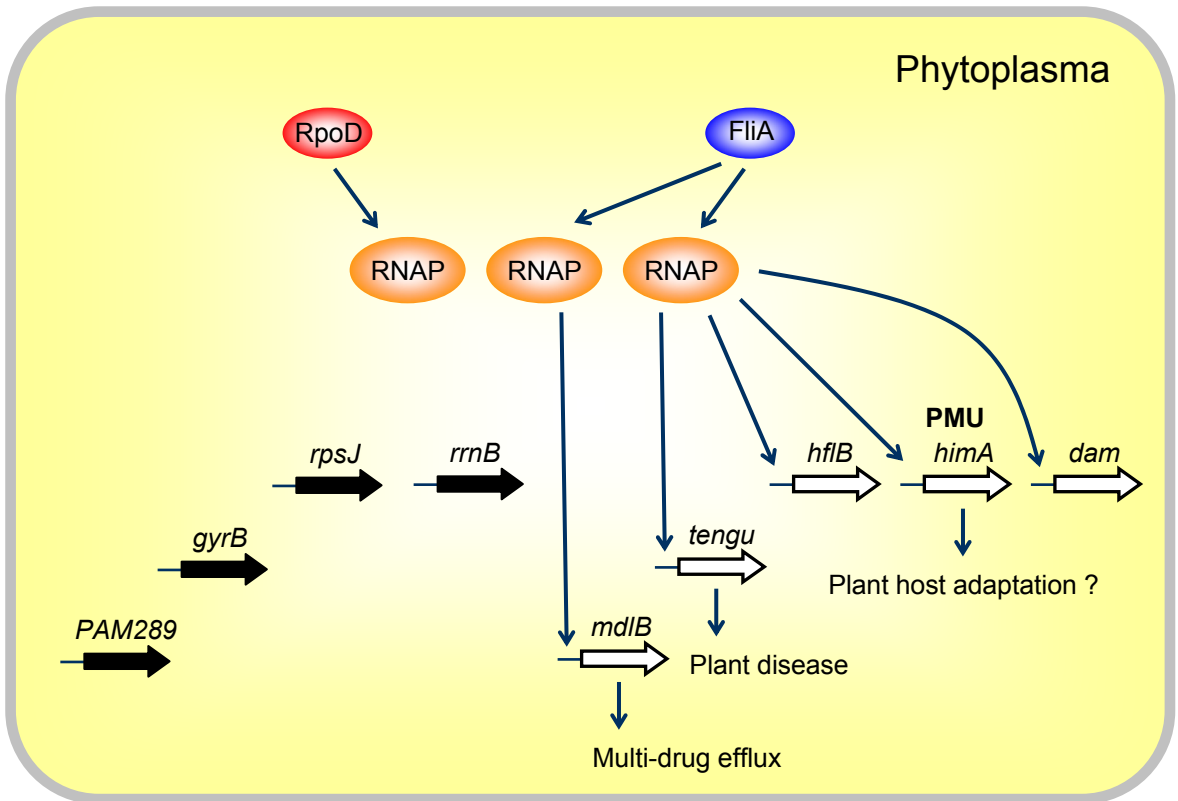

Supplement: Supplementary file 2 — Figure S1. and are indicated by lines above the alignment. Subregions 2.1 and 2.2 were reported to be involved in core binding, and subregions 2.4 and 4.2 were reported to be involved in promoter recognition Figure S2. Amino acid alignment of FliA. OY-M FliA (OY-M), Bacillus subtilis σF (Bacillus), Chlamydia trachomatis σ28 (Chlamydia), and Escherichia coli FliA (Escherichia) were aligned using the CLUSTAL W program. Amino acid similarity is indicated by highlighting (black shadow, >80% identity; gray shadow, 60–80% identity); gaps are indicated by hyphens. Several motifs (subregions) conserved among the many RpoD genes were defined based on previous studies (Lonetto et al. Figure S1. Amino acid alignment of RpoD. OY-M RpoD (OY-M), Bacillus subtilis σA (Bacillus), Chlamydia trachomatis σ70 (Chlamydia), and Escherichia coli σ70 (Escherichia) were aligned using the CLUSTAL W program. Amino acid similarity is indicated by highlighting (black shadow, >80% identity; gray shadow, 60–80% identity); gaps are indicated by hyphens. Several motifs (subregions) conserved among the many RpoD genes were defined based on previous studies (Lonetto et al.) and are indicated by lines above the alignment. Subregions 2.1 and 2.2 were reported to be involved in core binding, and subregions 2.4 and 4.2 were reported to be involved in promoter recognition Figure S3. A model illustrating the RpoD and FliA regulatory network in the OY-M bacterial cell during host adaptation. RpoD is significantly more abundant than FliA in insect hosts. RpoD binding to RNAP regulates the rrnB, rpsJ, gyrB, and PAM289 genes (filled arrows) that were highly expressed in insect hosts. RpoD and FliA exist in approximately equal amounts in plant hosts. FliA binding to RNAP regulates the mdlB, tengu, hflB, himA, and dam genes (open arrows) that were highly expressed in plant hosts. [file mbo30002-0553-sd2.pdf]
